# Natural Ventilation for Prevention of Airborne Contagion: Conclusions Overgeneralized

**DOI:** 10.1371/journal.pmed.0040189

**Published:** 2007-05-29

**Authors:** Hal Levin

This article and editor's summary give the impression that the tuberculosis infection rate was actually reduced by opening windows [1]. A careful reading of the article clearly states that while ventilation rates were measured, infection rates were merely calculated using the Wells-Riley equation. This is old news. While it is important to take into account the adequacy of the ventilation rate provided by mechanical ventilation systems, the ventilation rate through open windows is a function of window size, number, and location in a room as modified by indoor–outdoor temperature differences and wind direction and velocity.

Not every case will result in the differences observed in the Peruvian hospitals studied. One must be careful not to overgeneralize the results.

A new article, “Role of ventilation in airborne transmission of infectious agents in the built environment—A multidisciplinary systematic review” by Yuguo Li et al., is a thorough review of infectious disease transmission and ventilation just published in the February 2007 issue of the journal *Indoor Air*, the International Journal of Indoor Environment and Health, available at http://www.blackwell-synergy.com/toc/ina/17/1 [2].
